# Determination of iFGF23 Upper Reference Limits (URL) in healthy pediatric population, for its better correct use

**DOI:** 10.3389/fendo.2022.1018523

**Published:** 2022-11-09

**Authors:** Vincenzo Brescia, Antonietta Fontana, Roberto Lovero, Carmela Capobianco, Stella Vita Marsico, Tiziana De Chirico, Carla Pinto, Lucia Varraso, Angela Pia Cazzolla, Francesca Di Serio

**Affiliations:** ^1^ Clinical Pathology Unit, Azienda Ospedaliero-Universitaria (AOU) Policlinico Consorziale di Bari - Ospedale Giovanni XXIII, Bari, Italy; ^2^ Department of Clinical and Experimental Medicine, Università degli Studi di Foggia, Foggia, Italy

**Keywords:** fibroblast growth factor 23, Upper Reference Limits, pediatric, immunochemiluminescent, analitycal variability

## Abstract

**Background:**

The measurement of Fibroblast growth factor 23 (FGF23) may be useful in the diagnosis and management of abnormal phosphate metabolism in both patients with preserved renal function or with chronic kidney disease (CKD). FGF-23 tests differ considerably by molecule assayed (iFGF23 or cFGF23), analytical performance and reference ranges. We establish iFGF23 Upper Reference Limits (URL) in apparently healthy pediatric individuals using automated immunochemiluminescent assay.

**Methods:**

We measured the levels of plasma iFGF23 from 115 samples from apparently healthy pediatric subjects [59 (51.3%) individuals were male; median age 10 years (range 1–18)] included in an observational study conducted at Policlinico University Hospital of Bari. The method used for the iFGF23 assay was immunochemiluminescent sandwich assay developed by DiaSorin on the Liaison XL platform. Statistical calculation of 95% reference interval, right-sided (CLSI C28-A3) and verification of age and sex covariables was performed for the calculation of the URL.

**Results:**

The URL concentration of iFGF23 was 61.21 pg/mL (58.63 to 63.71, 90% CI). No significant differences were found between the median concentrations of iFGF23 differentiated by sex and age.

**Conclusions:**

The dosage of iFGF23 is important both for the differential diagnosis of the various forms of rickets, and for the subsequent monitoring of the effectiveness of drug treatment. We have established the URL for the iFGF23 Liaison test in apparently healthy pediatric subjects. The availability of iFGF23 pediatric reference values will allow a better clinical use of the test.

## Introduction

The fibroblast growth factor 23 (FGF23) is a peptide hormone belonging to the phosphatonin family, specific factors involved in the regulation of phosphate homeostasis (1). FGF23 is encoded by the PHEX gene located on chromosome 12 and is translated into a 251aa (32kDa) protein with a signal sequence of 24aa, a domain homologous to the other members of the FGF family, an N-terminal region of 155aa and a sequence of 72aa in the C-terminal domain. After cleavage of the 24aa signal peptide, a 227aa protein is secreted which represents the “intact” portion (iFGF23) with hormonal activity. iFGF23 can be cleaved between Arg179 and Ser180, by specific enzymes, leading to the formation of two inactive fragments: N-terminal and C-terminal. Detectable FGF23 molecules in plasma include the intact form and N- and C-terminal fragments. At concentrations above the reference values, the C-terminal fragment competes with the active molecule (iFGF23) for binding to the receptor complex and thus inhibits its biological function (2). FGF23 is secreted by osteoblasts and osteocytes and acts by binding, with low affinity, to specific receptors (FGFR1, 2, 3, 4) (1). Such receptors have an extracellular region with three immunoglobulin-like domains containing the binding site for the ligand, a transmembrane domain and a cytoplasmic domain containing tyrosine kinase. Receptors for FGF23 (FGFR) are ubiquitous; nevertheless, the action of FGF23 is mainly expressed at the renal and parathyroid level. In order to act, FGF23 must bind to its specific membrane receptor (FGFR1) in the presence of a co-receptor, a transmembrane protein called ‘Klotho’ (3). Klotho is expressed mainly in the kidney, parathyroid and choroid plexus and makes the action of FGF23 organ specific. FGF23 reduces the proximal tubular reabsorption of phosphorus, by inhibiting the sodium co-transporters Npt2a and Npt2c, in order to maintain a neutral balance and avoid the accumulation of this cation and the appearance of hyperphosphorus (4). Activation of the FGF23/Klotho axis reduces the conversion of native vitamin D 25 hydroxylated (25 (OH) D) to its active form 1,25dhydroxyvitaminD (1,25 (OH) 2D) through inhibition of the expression of 1 -alpha-hydroxylase and promotes the degradation of vitamin D (1,25 (OH) 2D) by inducing 24-hydroxylase (5).

In patients with iron deficiency or with acute inflammatory processes, an increased production of FGF23 was detected simultaneously with an increase in the cleavage of the same molecule; in this case a greater quantity of fragments was found in the plasma than in intact molecules (6). In patients with Chronic kidney disease-mineral and bone disorder (CKD-MBD) there is a down-regulation of the cleavage mechanism so that most of the circulating molecules are iFGF23 (7).

Measurement of FGF23 may be useful in the diagnosis and management of phosphate metabolism abnormalities among patients with preserved renal function or overt renal insufficiency. FGF23 plays a fundamental role in maintaining the phosphorus balance in CKD (state of excess phosphorus) with a subsequent decline of 1,25 (OH) 2D and could underlie the onset of secondary hyperparathyroidism (HPT) (8).

FGF23 has been proposed as a promising biomarker for the prediction of adverse outcomes, including cardiovascular disease (CVD) and mortality, in patients with chronic renal failure. Elevated FGF23 levels are related to the development of coronary artery disease (CAD) and left ventricular hypertrophy (LVH) and myocardial ischemia (MI), stroke, and impaired immune response (9, 10). X-linked hypophosphatemia (XLH) is the most common genetic form of hypophosphatemic rickets and osteomalacia. In this disease, mutations in the PHEX gene lead to elevated levels of FGF23 resulting in renal loss of phosphate and impaired skeletal and dental mineralization. Recently, international guidelines for the diagnosis and treatment of this condition have been published (11, 12) Two main types of enzyme immunoassays are currently available for the determination of FGF23 in human plasma or serum: intact FGF23 test (iFGF23) which detects only full-length FGF23 by simultaneous recognition of epitopes on the N- and C-terminal domains near the proteolytic cleavage site; C-terminal FGF23 (cFGF23) which detects both iFGF23 and C-terminal fragments with two antibodies against two epitopes within the C-terminal portion of the FGF23 and C-terminal molecule (13–15). All commercially available tests for the assay of FGF23 differ considerably from each other in that they use antibodies targeting distinct epitopes on the protein, different calibrators, and are not harmonized. In addition, the iFGF23 and cFGF23 assays present differences in analytical performance, in the intra and intersection coefficient of variation, in accuracy, in reproducibility with different sensitivities (16–18). (19). iFGF23, measured using an automated platform, can improve the performance characteristics and clinical use of this molecule. However, the absence of work on the reference values of iFGF23 in the pediatric population limits the use of the molecule for a correct interpretation of calcium-phosphate disorders and the onset of bone disorders in early stages or in asymptomatic subjects at risk (20)

The aim of our study was to verify the high reference limit (URL) of iFGF23 in a group of apparently healthy pediatric subjects measured on an automated platform with immunochemiluminescent method.

## Materials and methods

### Study subjects

This observational study was approved by ethical committees of the Policlinico University Hospital of Bari (Biomarkers of Bone Metabolism; Study number. 38359/COMET of 27 April 2021 BMOPed). This study was conducted in accordance with the principles of the Declaration of Helsinki and the International Conference on Harmonization Guidelines for Good Clinical Practice.

The samples were collected from 120 healty subjects (58 women and 62 men; age range, 1-18 years; mean age 10.87 years (9.96 to 11.78, 90% CI) median age 11.5 years (9.9 to 13.0, 90% CI). Apparently healthy children were enrolled in the study and signed informed consent was obtained from parents, in addition they had a negative history of chronic disease, kidney disease, heart disease, gastrointestinal disease, endocrine disorders or minor surgical procedures and other conditions such as pregnancy, which would have affected the calcium-phosphate balance.

Inclusion criteria were a normal physical work-up (weight, height, nutritional status and gonadal/sexual status), normal laboratory values determined after an overnight fast (serum sodium, potassium, calcium, phosphate, creatinine, glicemia, liver enzymes, TSH, FreeT4, blood cell counts, albuminemia, iron, transferrin, ferritin, C-reactive protein (CRP), The exclusion criteria were thyroid, renal, hepatic, cardiovascular, pulmonary, intestinal pathologia or with clinically significant disease entities who were hospitalized in our center for diagnostic reasons severe illness occurring or surgical procedures including bone fracture within 3 months. Children with abnormal laboratory results and/or with vitamin D deficiency status based on circulating levels <50 nmol/L (<20 ng/ml).

### Analyzer

The level of iFGF23 in plasma was evaluated with the LIAISON^®^ FGF 23 kit. The test is a new automated immunochemiluminescent sandwich assay (CLIA) developed by DiaSorin (Saluggia, Italy) on the Liaison XL platform for the quantitative *in vitro* assay of the intact polypeptide FGF23 in EDTA plasma samples. The analytical method is based on a sandwich test using three different antibodies. A monoclonal antibody adhered to magnetic microparticles (solid phase) and directed against the N-terminal portion of the FGF23 molecule; another monoclonal antibody directed against a different epitope (C-terminal portion of the FGF23 molecule) labeled with fluorescein, and the third monoclonal antibody, bound with isoluminol and directed against fluorescein. The light signal is proportional to the amount of antibody-isoluminol conjugate and is indicative of the concentration of iFGF23 present in the calibrators, samples or controls. The instrument directly calculates the concentration of iFGF23 expressed in pg/mL.

According to the manufacturer’s report, the Limit of Detection (LoD) was 5 pg/mL; the limit of quantification (LoQ) was 6.5 pg/mL. CV repeatability (%) ranged from 2.6 to 5.3% depending on the different analyte concentrations in the samples. The manufacturer’s declared reference range was 23.2–95.4 pg/mL (2.5–97.5 percentile), referring to a healthy adult population.

### Analyte determination

The list of analytes measured for the selection of subjects; the reference intervals are differentiated by age and sex are in **Table 1**. Dosage of 25 (OH) Vitamin D was performed with chemiluminescence assay using the TGSTA Technogenetics instrumentation (Technogenetics, Milano-Italy). Serum calcium, phosphorus, iron, glucose, albuminemia, was carried out using the spectrophotometric method; bichromatic endpoint technique (polychromatic albumin andpoint); serum sodium, potassium, was carried out using potenziomatric method, creatinine (coupled enzymatic) liver enzyme (AST, ALT) was carried out by enzyme method (bichromatic kinetic technique); TSH, FreeT4, ferritin was carried out by chemiluminescente methodica (LOCI) Transferring (immunoenzymatica) and PCR CRP was carried out by nephelometric method. These analytes were assayed on These following analytes were assayed on Dimension VISTA 1500 instrumentation (Siemens, Munich, Germany). count The samples were collected in tubes with EDTAK3 anticoagulant for blood count and analyzed on ADVIA 2120i hematology analyzer.

**Table 1:** Descriptive statistics of the distribution of the values of the analytes assayed.

Analyte	Reference value	Minimum	Maximum	Mean
**1,25(OH)2 vitamin D** (pg/mL)	15.2-90.1	44,30	88,60	62,92
**25OH vitamin D** (ng/mL)	>20	20,00	27,00	22,95
**serum calcium** (mmol/L)	2,38-2,82 (0-2 y)	2,39	2,545	2,44
** **	2,37-2,69 (2-5 y)	2,34	2,545	2,445
** **	2,28-2,55 (5-19 y)	2,19	2,545	2,368
**Serum phosfates** (mmol/L)	1,42-1,99 (M; 1-4y)	1,41	1,73	1,556
	1,29-1,84 (M; 5-12y)	1,15	1,82	1,454
	1,05-1,82 (M; 13-15y)	1,12	1,63	1,366
	0,87-1,57 (M; 16-19y)	1,12	1,6	1,375
	1,42-1,99 (F; 1-4y)	1,41	1,66	1,504
	1,29-1,84 (F; 5-12y)	0,99	1,54	1,348
	1,05-1,68 (F; 13-15y)	1,09	1,6	1,246
	0,87-1,57 (F; 16-19y)	0,83	1,57	1,248
**Serum creatinine** (mg/dL)	0,2-1,2	0,21	0,89	0,53
**Estimated glomerular filtration rate eGFR** (mL/min/1,73m^2^)	>60	95,00	232,00	157,31
**FT4** (ng/dL)	0.76-1.48 8	0,73	1,75	1,03
**TSH** (mUI/L)	0,66-3.98	0,80	3,22	2,09
**Serum albumin **(g/dL)	3,8-5,6	3,30	4,70	4,09
**Serum glucose** (mg/dL)	<100	49,00	98,00	80,27
**Serum potassium (K)** (mmol/L)	3,7-4,8	3,60	6,00	4,33
**Serum sodium (Na)** (mmol/L)	134-143	4,20	142,00	135,45
**White blood cells (wbc)** x103/mL	5,5-11,0	5,53	10,20	6,82
**C reactive protein (CRP)** (mg/L)	<3,0	<2,9		
**Hemoglobin (Hgb)** (g/dL)	11,3-14,5	10,90	14,19	13,40
**Serum Iron** (µg/dL)	23-203	11	165	75,459
**Serum Transferrin** (mg/dL)	200-360	143	364	269,81
**Serum Ferritin** (ng/mL)	26-388	9	189	40,351

Renal function was assessed based on creatinine levels and estimated glomerular filtration rate (eGFR) with Schwartz equation [k × height (cm)/serum creatinine], with different k values for age and sex (k = 0.450, 0.55, 0.7 for infants aged less than 12 months, <12 years and children ≥13 years, respectively).

### Collection and storage of samples

Blood samples were collected between January and April 2022. Fasting and morning samples were performed to measure iFGF23 levels in sterile EDTA vacutainers, centrifuged at 3000 × g, 10 min, 4° C. Plasma was removed and aliquoted into polypropylene tubes for storage at -80° C pending testing. Before storage the samples were evaluated for interference from hemolysis (hemoglobin> 1,000 mg/dL), jaundice (conjugated and unconjugated bilirubin greater than 20 mg/dL) and chylosity (triglyceride concentrations> 3,000 mg/dL) the HIL method [Dimension VISTA 1500 instrumentation (Siemens, Munich, Germany)]. The sample was thawed at room temperature prior to analysis.

### Verification of the analytical quality of the method according to iFGF23

Interassay analytical variability (CVA) was assessed using internal quality control materials on two concentration levels (Level 1 Control and Level 2 Control) provided by the kit manufacturer according to the CLSI EP15A2 standard procedure (five-day evaluation with intake in triplicate) (21) The CVA obtained was compared with the analytical quality objectives referred to biological variability (16) and with those provided by the supplier [LIAISON^®^ FGF 23 (REF. 318700) 6/11 IT-200/007-061, 05-2019 -09].

Evaluation of accuracy required performing a dilution (or parallelism) test. Three samples at different concentrations were used for the test (sample A: 210 pg/mL; sample B: 365 pg/mL; sample C: 7189 pg/mL); the dilution was performed using a specific diluent (LIAISON^®^ FGF 23). The recovery percentage was calculated by comparing the measured iFGF23 concentrations with those expected (test acceptability criterion: 90-110%).

### Statistical analyzes

For the calculation of the reference values only an upper limit of normality (URL) was used as only high values are to be evaluated in case of suspected pathology. Mean and median iFGF23 concentrations and the standard deviation (SD), distribution intervals [5th –95th percentile and 90% confidence intervals (CI)] were calculated using standard parametric and non-parametric statistical analyzes. Distribution normality was evaluated using the D’Agostino-Pearson Test. We removed outliers in partitions with normally distributed data using the Tukey test; 95% reference interval, right-sided, was calculated according to the guidelines (CLSI C28-A3) with parametric percentile method (22, 23). Scatterplots were created to visually inspect the data (24). The frequency histogram was produced to have a visual interpretation of numerical data by showing the number of concentrations that fall within a specified range of values.

Centiles (0.025; 0.05; 0.95; 0.975) at different ages were evaluated to verify the distribution of iFGF23 in the evaluated pediatric population.

The measurements are modeled on age using weighted polynomial regression (Altman & Chitty, 1994). This regression model gives the mean of the measurements as a function of age: mean (age). Scatter plot was used to visualize the distribution of the measurements versus age with evidence of the calculated mean (central line) and centile curves.

In addition, the graph showing the z-scores plotted against age was produced. According to the distribution of the z-score values, the correct selection of the iFGF23 data, without outliers, is indicated by the evidence that less than 5% of the values obtained were above and below the line corresponding to z = +/-1.645.

Mann-Whitney U test has been used to evaluate whether iFGF23 levels stratified by gender and age were from the same population. A P-value <0.05 was considered statistically significant.

Multiple comparison procedures (pairwise comparison) was used in an ANOVA test to compare the means obtained in the age-stratified subgroups. The mean difference, P-value and 95% Confidence Interval of the difference is given. If the P-value was low (P <0.05) it can be concluded that there was significant difference between measurements.

The degree of relationship between iFGF23 towards variables 1-25 (OH) 2D, 25 (OH) D, total calcium and phosphate was determined using rank correlation. The relationship between two numerical variables was presented graphically in the scatter plot with trend line and color map, the background color coding indicated the density of the points.

The MedCalc^®^ (11.6.1.0) program was used for statistical analyzes.

## Results

120 samples were collected for the iFGF23 assay from apparently healthy pediatric subjects, 115 samples were included while 5 samples considered outliers were excluded based on the evaluation with the Tukey test. The outliers samples did not belong to a single age-stratified group of subjects. In the group of subjects evaluated 59 (51.3%) individuals were male and 56 (48.7%) were female, the mean age was 10.71 years (9.78 to 11.64, 95% CI). All samples included in this study had analyte values assayed within the limits of the reference ranges stratified by gender and age (**Table 1**).

### Verification of method analytical variability

The repeatability study according to the EP15A2 standard provided CV (%) values of 2.5 (SD 5.30 pg/mL; 1.50 to 7.20 95% CI) and CV (%) 2.10 (SD 13.98 pg/mL; 1.30 to 6.10 95% CI) for concentration levels of 213.50 pg/mL and 650.98 pg/mL of iFGF23, respectively. These CV values (%) met the desired quality specifications assessed on the basis of biological variability data [3.9 CV (%) based on CVI (%)] (16) and were comparable with the data reported by the supplier [CV (%) 2.6 and 2.7 (LIAISON^®^ FGF 23 (REF. 318700)]

The study of the percentage recovery by dilution test showed percentage deviations greater than ± 10%, from the expected value, for dilutions greater than 16 and the expected concentration close to the Limit of Quantitation (LoQ) of 6.5 pg/mL (**Table 2**).

**Table 2:** The concentrations of the samples, the dilutions used and the recovery percentage obtained (acceptability between 90 and 110%) with dilution test are reported.

Sample iFGF23	Dilutions	Expected value (pg/mL)	Measured value (pg/mL)	Recovery(%)
**A**	2	105,00	93,50	90,95
(210 pg/mL)	4	52,50	55,75	106,19
	8	26,25	28,50	108,57
	16	13,13	14,01	106,74
	32	6,56	19,78	301,43
**B**	2	182,50	195,65	107,21
(365 pg/mL)	4	91,25	86,23	94,49
	8	45,63	44,61	97,78
	16	22,81	21,77	95,42
	32	11,41	12,47	109,32
**C**	2	3594,50	3619,50	101,28
(7189 pg/mL)	4	1797,25	1790,25	99,61
	8	898,63	829,00	92,25
	16	449,31	410,63	91,39
	32	224,66	229,22	102,03
	50	143,78	150,86	104,92
	100	71,89	97,91	136,19

### Calculation of Upper Reference Limit

In the 115 samples evaluated the value of iFGF23 was 22.99 and 70.00 pg/mL respectively in the sample with the lowest and highest concentration. The mean obtained was 45.16 pg/mL and the median was 44.10 pg/mL with a normal distribution of values (D’Agostino-Pearson test).

The URL concentration of iFGF23 obtained as 95% Reference range, right side parametric percentile method (according to CLSI C28-A3) was 61.21 pg/mL (58.63 to 63.71, 90% CI)

The descriptive statistical analysis of the distribution of the samples before and after the exclusion of the outlier values has been reported in **Table 3**. The differences in the distribution of the values according to the presence or exclusion of the outlier values are evident.

**Table 3:** The descriptive statistical analysis of the samples included in the study and the URL calculation of iFGF23 on 115 pediatric samples distributed by gender.

Measurements	Total values with outliers	Total values without outliers	iFGF23 in Female	iFGF23 in Male
**Sample size** (n.)	120	115	56	59
**Lowest value** (pg/mL)	14,20	22,99	22,99	25,55
**Highest value** (pg/mL)	87,17	70,00	70,00	67,73
**Arithmetic mean** (pg/mL)	45,58	45,16	45,99	44,36
**Median** (pg/mL)	44,52	44,10	45,09	42,71
**Standard deviation** (pg/mL)	11,99	9,93	9,66	10,20
**D'Agostino-Pearson test for** **Normal distribution**	reject Normality (P=0,0041)	accept Normality (P=0,5466)	accept Normality (P=0,5065)	accept Normality (P=0,5168)
**Tukey test for outliers: values**(pg/mL)	14,2; 16,6; 75,75; 82,76; 87,17	none	none	none
**Upper Reference Limit (URL)** (pg/mL) **90% CI** (pg/mL)	not evalueted	61,21 *58,63 to 63,71*	61,79 57,92 to 65,45	61,30 57,42 to 64,68

**Figure 1:**
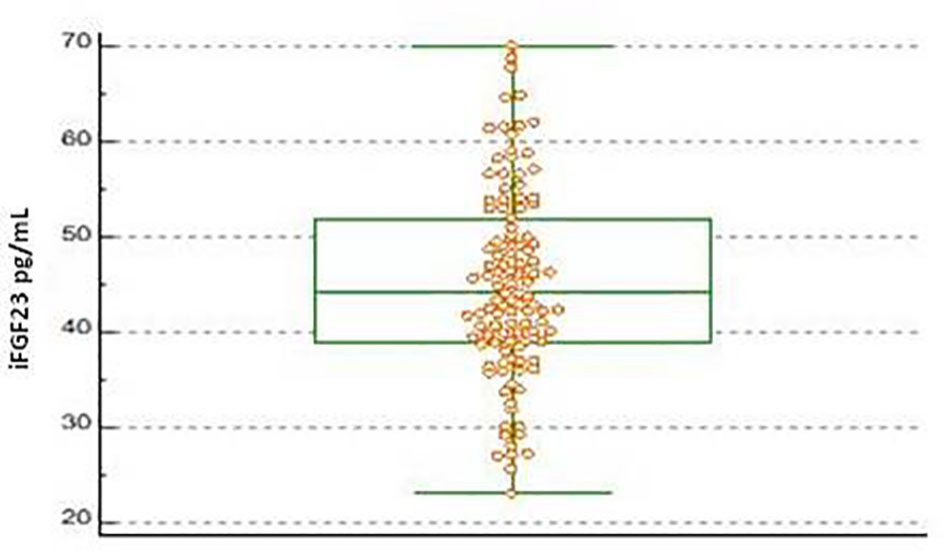
Boxplot of iFGF23 concentration obtained on 115 pediatric subjects evaluated. The central box shows the values from the 25th to the 75th quartile, the central line the median, the horizontal lines the extension from the minimum to the maximum value (range).

**Figure 2:**
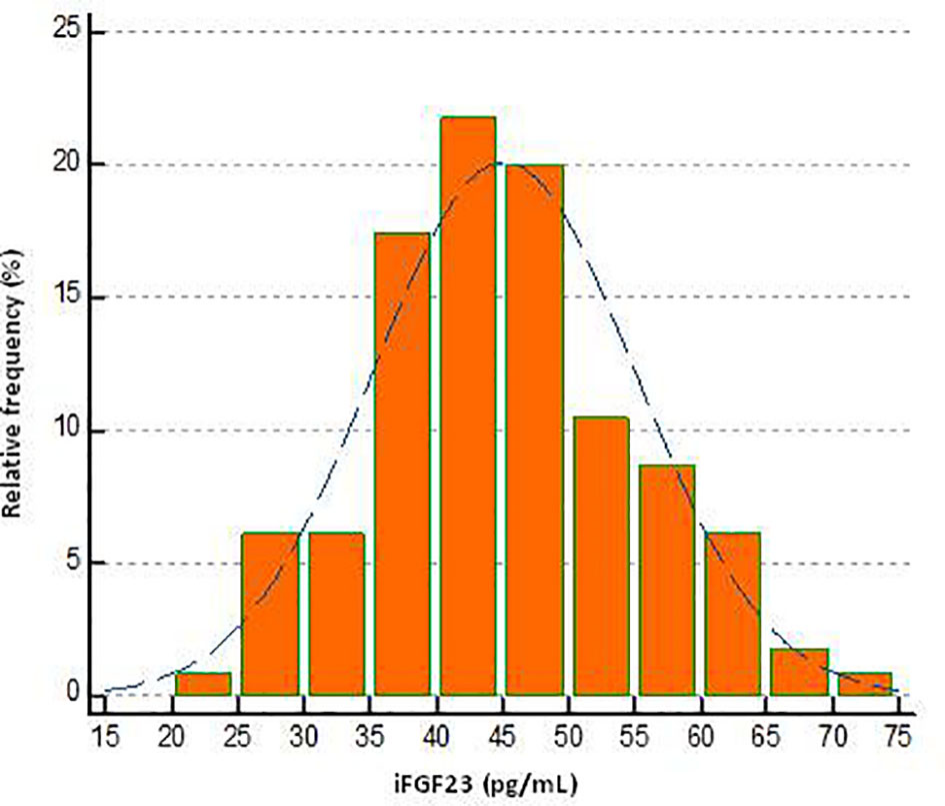
Frequency histogram of iFGF23 concentration in pediatric subjects evaluated.

The scatterplots obtained from the distribution of iFGF23 values in the pediatric subjects included for the calculation of the URL were reported in **Figure 1**. No suspected outlier was highlighted. The frequency distribution histogram (%) showed the distribution of the samples and the underlying normality curve (**Figure 2**).

### Evaluation of covariables

All iFGF23 values were grouped into 9 age groups and centile values were provided for each. The data obtained were reported in **Table 4**. The age group of 2 years and 18 years showed a dispersion of values at 0.025-0.975 centiles, respectively, minimum (range 29.29 to 61.23 pg/mL) and maximum (range 22.64 to 66.85 pg/mL).

**Table 4:** The centiles calculated for different ages are shown.

Variable	Centiles of iFGF23 (pg/mL)			
Age (years)	0.025	0.05	0.95	0.975
2	29,29	31,86	58,66	61,23
4	27,73	30,51	59,63	62,42
6	25,21	28,28	60,27	63,33
8	22,59	25,92	60,70	64,03
10	20,70	24,22	61,04	64,57
12	20,37	23,95	61,40	64,98
14	22,45	25,89	61,89	65,33
16	27,77	30,81	62,62	65,67
18	22,62	42,28	66,85	67,73

**Figure 3:**
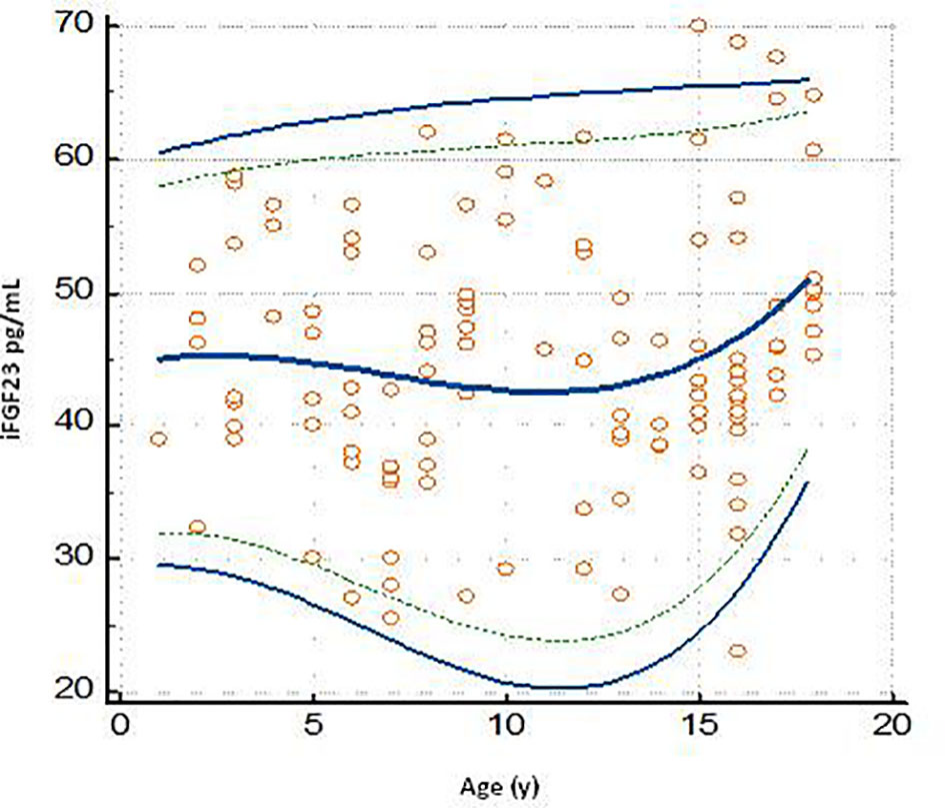
Plot of the scatter diagram of the measurements of iFGF23 versus age. The graph also shows the calculated mean (central line) and centile curves (solid line: 0.025-0.975 centiles; broken line: 0.05-0.95 centiles) to the individual measurements.

**Figure 4:**
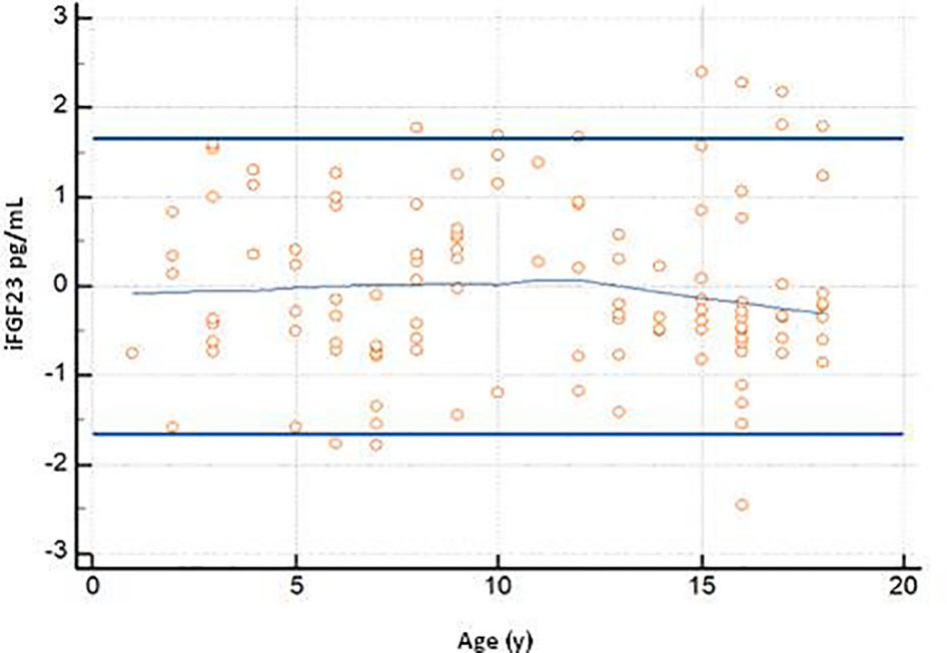
The graph shows the z-scores plotted against age Horizontal lines are drawn at z-scores of -1.645 and 1.645. At the center of the trendline.

The graph of the dispersion diagram of the measurements with respect to age had not highlighted groups of subjects with an apparent different distribution of the iFGF23 values (**Figure 3**). The z-scores showed no pattern and are randomly scattered across all ages. It was found that less than 5% of cases were above the line corresponding to z = 1.645 and less than 5% of cases were below the line corresponding to z = -1.645 (**Figure 4**).

In order to evaluate any significant changes in the concentration of iFGF23, three age limits were arbitrarily identified (1) 1-6 years, (2) 7-12 years and (3) 13-18 years.

The Mann-Whitney test revealed no significant differences (P> 0.05) between the median concentrations of iFGF23 in the three age groups of pediatric subjects. The test results are shown in **Table 5**.

**Table 5:** Comparison of the difference between the medians in the groups of subjects stratified by age and sex (Mann-Whitney test).

Variable	Two-tailed probability
iFGF23 (1-6 years) vs iFGF23 (7-12 years)	P = 0,5183
iFGF23 (1-6 years) vs iFGF23 (13-18 years)	P = 0,7562
iFGF23 (7-12 years) vs iFGF23 (13-18 years)	P = 0,3404
iFGF23 Males vs iFGF23 Females	P = 0,3429
iFGF23 (1-6 years) Females vs iFGF23 (7-12 years) Females	P = 0,5050
iFGF23 (1-6 years) Females vs iFGF23 (13-18 years) Females	P = 0,1967
iFGF23 (7-12 years) Females vs iFGF23 (13-18 years) Females	P = 0,9199
iFGF23 (1-6 years) Males vs iFGF23 (7-12 years) Males	P = 0,2335
iFGF23 (1-6 years) Males vs iFGF23 (13-18 years) Males	P = 0,6888
iFGF23 (7-12 years) Males vs iFGF23 (13-18 years) Males	P = 0,4450

The significant Multiple comparison procedures ANOVA test result (P> 0.05) suggested that the means obtained in the age-stratified subgroups were the same across the groups being compared (**Table 6**).

**Table 6:** Multiple comparison procedures (pairwise comparison) in ANOVA test in age-stratified subgroups.

Factors	Mean difference	P	95% CI
iFGF23 (1-6 years) vs iFGF23 (13-18 years)	2,427	1	-4,477 to 9,331
iFGF23 (1-6 years) vs iFGF23 (7-12 years)	2,952	0,7226	-3,321 to 9,225
iFGF23 (13-18 years) vs iFGF23 (1-6 years)	-2,427	1	-9,331 to 4,477
iFGF23 (13-18 years) vs iFGF23 (7-12 years)	0,525	1	-6,572 to 7,622
iFGF23 (7-12 years) vs iFGF23 (1-6 years)	-2,952	0,7226	-9,225 to 3,321
iFGF23 (7-12 years) vs iFGF23 (13-18 years)	-0,525	1	-7,622 to 6,572

**Figure 5:**
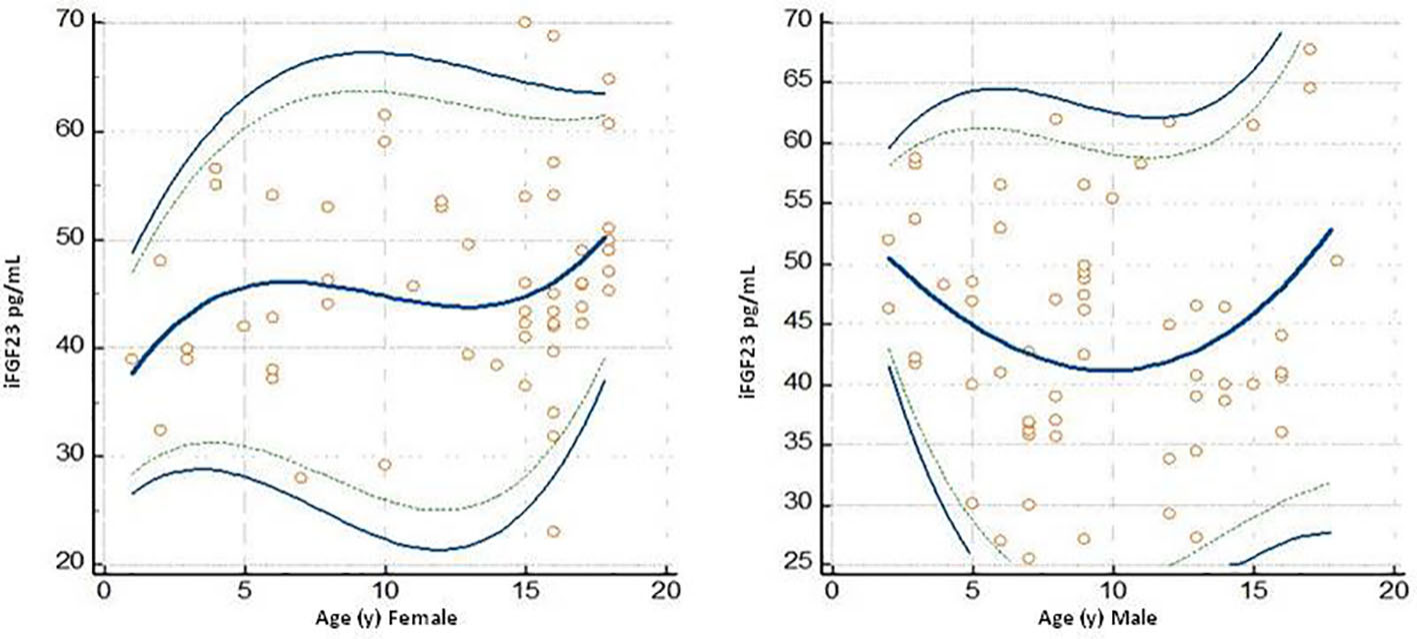
Plots dello scatter diagram of the measurements versus sesso (Female and Male) ed age I grafici riportano olte alle singole misurazioni the calculated mean (central line) and centile curves (linea continua 0,025 0,975 centiles, linea discontinua: 0,05-0,95 centiles).

In the 115 samples evaluated, the median concentrations of iFGF23 in the female versus male subjects were 45.09 pg/mL and 42.71 pg/mL, respectively. The URL concentration of iFGF23 in female subjects was 61.79 pg/mL (57.92 to 65.45 pg/mL; 90% CI) and in male subjects it was 61.30 pg/mL (57.42 to 64.68 pg/mL; 90% CI). The data are shown in **Table 3**. The graph of the dispersion diagram of the measurements with respect to age in the two groups of subjects differentiated by sex had not shown a different distribution of the obtained values of iFGF23 (**Figure 5**)

Furthermore, there were no significant differences (P> 0.05) between the median concentrations of iFGF23 in the three groups of pediatric subjects identified by age and differentiated by sex (Mann-Whitney test) (**Table 5**).

**Figure 6:**
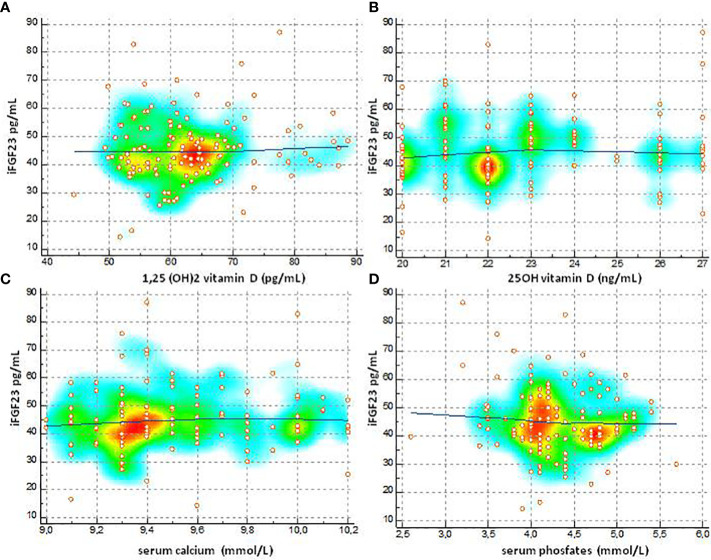
Degree of relationship between IFGF23 towards variables 1-25 (OH) 2D, 25 (OH) D, total calcium and phosphate **(A)** Spearman's (rho) 0.0489, 95% CI for rho -0.132 to 0.226, P=0.5960, **(B)** Spearman's (rho) 0.104, 95% CI for rho 0.0770 to 0.278, P = 0.2597, **(C)** Spearman's (rho) 0.093, 95% CI for rho -0.0967 to 0 276, P = 0.3359, **(D)** Spearman's (rho) -0.787, 95% CI for tho -0.254 to 0102, P=0.3931].

The degree of relationship between iFGF23 towards variables 1-25 (OH) 2D, 25 (OH) D, total calcium and phosphate was shown in **Figure 6**. The values of Spearman’s rank (rho) correlation coefficient, the interval 95% confidence, the associated P value indicated that no significant relationship was evident between the variables. The graphical presentation with a color-map scatter plot confirmed the results.

## Discussion

FGF23 is of great importance, both for the differential diagnosis of the various forms of rickets, and for the subsequent monitoring of the effectiveness of drug treatment. There are several commercially available enzyme immunoassays that measure different fractions of FGF23 (cFGF23 and iFGF23) in serum or plasma (25, 26). The data in the literature confirm that the dosages of FGF23 differ by method, by compatibility with the different automated platforms, by expression of the reference intervals (RI) and also in the measurement units (27, 28). Therefore, it is of fundamental importance to have in the laboratory verified reference values based on physiological variations and the method used. CLSI has produced document C28-A3 containing guidelines for verifying reference ranges. The standard requires that suitable pre-analytical procedures (collection, transport and handling of the sample) are used and that the analytical specificities within the laboratory are verified (22). To avoid a reduction of iFGF23 in the sample due to fragmentation, a collection of blood anticoagulated with ethylenediaminetetraacetic acid (EDTA) was used, as iFGF23 is significantly more stable in plasma than in serum, and centrifugation was performed within 2 hours of execution of the sample (29–31). Furthermore, only morning fasted samples were evaluated to avoid diurnal variations in iFGF23 concentrations (19, 28, 32, 33). In accordance with the CLSI standard to increase the reliability of the values to be included in the statistical analysis of the calculation of the reference values, the analytical performance of the Liaison iFGF23 test was verified by testing the coefficient of variation (CV) and the linearity of the dilutions serial. The imprecision values we obtained were consistent with the analytical quality objectives (CV) calculated on the basis of the biological variability (CVI) of iFGF23 (16), the CV provided by the manufacturer and the CV reported by Souberbielle et al. (19) who used our assay method. The recovery percentage was suitable with dilution up to 16, well above the dilution factor 10 recommended by the method, with the possibility of measuring samples with iFGF23 concentrations higher than the analytical range of 5000 pg/mL. This is particularly useful in hemodialysis patients who often have iFGF23 concentrations above the functional test range. In fact, N. Yamamura-Miyazaki reported iFGF23 values increased by 10 times the maximum detection limit in pediatric subjects with CKD stage 5 and measured with the ELISA method (ELISA kit, Kairos; anal range 3-800 pg/mL) (34).

URL for iFGF23 evaluated on the total of 115 pediatric subjects included in our study was 61.21 pg/mL (58.63 to 63.71 pg/mL, 90% CI) and obtained with an analytical method with analytical sensitivity of 5 pg/mL. Concentrations of iFGF23 were evaluated in categories of subjects stratified by age and sex to verify if there were changes in concentration related to hormonal changes that occur during pubertal growth and development. The data reported in the literature on the dependence of FGF23 at age and sex are sometimes inconsistent. In general, studies indicate that cFGF23 may be age- and sex-dependent and may express higher values in early childhood and adolescence (25, 33, 35–39), on the other hand iFGF23 does not change according to age (33–40).

Our results on iFGF23 showed no differences in concentrations. in the different age groups and the tendency to have higher values in girls in the younger (F age 1 -6 years) and older (F age 13 -18) group, was not statistically significant.

FGF23 is an expression of bone metabolism and its concentration could vary according to the calcium-phosphate levels and with the concentration of vitamin D. FGF23 is a phosphatic hormone so it is conceivable that lower serum phosphates can be associated with levels of FGF23 higher. Our study found that iFGF23 values in childhood and adolescence are not affected by phosphate and calcium concentrations (phosphate r = -0.0787, P = 0.39) (total calcium r = 0.0931, P = 0.33). In healthy children, despite the age-related variation in serum phosphate levels, no correlation with iFGF23 was found. The precise mechanism by which physiological changes in serum phosphate concentrations are not associated with changes in FGF23 synthesis is unclear (39, 41–43). Obviously, this is not valid in the population with chronic renal failure since in these patients FGF23 acts by protecting the body against excessive phosphate load (39, 44). The relationship between expression of FGF23 and concentrations of 1,25- (OH) 2D (5, 45, 46) is known. The pediatric subjects selected for our study did not have severe vitamin D insufficiency and baseline values of 1.25- (OH) 2D and 25 (OH) D did not correlate with baseline iFGF23 (1.25 (OH) 2D) levels. r = 0.048, P = 0.5960) (25 (OH) D r = 0.104, P = 0.2597).

Many clinical disorders can be associated with changes in FGF23 concentrations. In patients with early stages of chronic kidney disease, FGF23 levels begin to increase early with eGFR values below 90 ml/min/1.73 m2 in both the adult and pediatric population (20, 34, 47, 48). Our study enrolled children whose eGFR was> 90ml/min/1.73m; therefore children with potential mild renal dysfunction and possible overestimation of iFGF23 were excluded. Iron deficiency can cause increases in FGF23 levels (6, 49). The pediatric population and adolescent girls can be considered at risk for iron deficiency; Malgorzata S. et al. hypothesized that the tendency to higher values of FGF23 found in girls could hypothetically be explained by the association between FGF23 and the sideremic state (40). Our study included pediatric subjects who presented a suitable martial attitude, therefore the considerations on the non-significant variations of iFGF23, dependent on age and sex, and the association with the state of iron were only hypothetical. Some studies report the increase of FGF23 in subjects with inflammatory diseases (eg, acute inflammation/sepsis, rheumatoid arthritis and childhood inflammatory bowel disease) (50–52). The mechanisms underlying this relationship are unclear; however, it has been suggested that inflammation stimulates the expression of FGF23 in osteocytes which promote the production of inflammatory cytokines in the liver (6, 34). In our study, apparently healthy subjects who did not show changes in biochemical data, in particular CRP, indicators of inflammation, were enrolled.

Having reference values of method-dependent iFGF23 is fundamental as some guidelines, approved by the competent national authorities, specify that FGF23 can be dosed at starting during therapy with burosumab, anti-FGF23 antibody, in the X-linked** **hypophosphatemia (XLH) but report decision values (RI) specific to an assay method (53). The lack of pediatric reference intervals, method dependent, the evidence of insufficient standardization and harmonization tests and knowledge of possible interferences in the assay, make the iFGF23 ed la conoscenza di possibili interferenze nel dosaggio, make the iFGF23 measurements not validated in the therapy of XLH (54, 55). Ours was one of the first papers to report iFGF23 concentrations in the pediatric population using the recently commercialized iFGF23 (Diasorin) kit. To this end, we have applied recognized analytical validation procedures and calculation of reference values using samples of pediatric subjects recruited according to particularly selective criteria.

The limit of our study was the number of subjects included in the various subgroups. There is an objective difficulty in finding suitable biological material in apparently healthy subjects in pediatric age. Long-term observational studies at multiple centers using the same assay method and capable of providing suitable analytical performance could further improve knowledge on iFGF23.

## Conclusions

We established the URL for the iFGF23 Liaison test from a well-defined cohort of apparently healthy pediatric and adolescent subjects. The fully automated chemiluminescence method used for iFGF23 demonstrated optimal analytical characteristics and a wider analytical range than ELISA kit-based methods.

## Data availability statement

The raw data supporting the conclusions of this article will be made available by the authors, without undue reservation.

## Ethics statement

The studies involving human participants were reviewed and approved by 38359/COMET. Written informed consent to participate in this study was provided by the participants’ legal guardian/next of kin.

## Author contributions

Concept and design: VB and AF. Acquisition, analysis, and interpretation of data: CP, RL and SVM. Drafting of the manuscript: TDC, LV, CC. Bibliographic research: AF, LV, APC and CC. Critical revision of the manuscript for important intellectual content: FDS, VB, APC. Data interpretation, technical, and material support: VB and RL. Supervision and final approval: VB, FDS. All authors contributed to the article and approved the submitted version.

## Conflict of interest

The authors declare that the research was conducted in the absence of any commercial or financial relationships that could be construed as a potential conflict of interest.

## Publisher’s note

All claims expressed in this article are solely those of the authors and do not necessarily represent those of their affiliated organizations, or those of the publisher, the editors and the reviewers. Any product that may be evaluated in this article, or claim that may be made by its manufacturer, is not guaranteed or endorsed by the publisher.
